# DNA reference libraries of French Guianese mosquitoes for barcoding and metabarcoding

**DOI:** 10.1371/journal.pone.0176993

**Published:** 2017-06-02

**Authors:** Stanislas Talaga, Céline Leroy, Amandine Guidez, Isabelle Dusfour, Romain Girod, Alain Dejean, Jérôme Murienne

**Affiliations:** 1 Institut Pasteur de la Guyane, Unité d’Entomologie Médicale, Cayenne, France; 2 UMR EcoFoG, CNRS, AgroParisTech, Cirad, INRA, Université de Guyane, Université des Antilles, Kourou, France; 3 IRD, UMR AMAP (botAnique et Modélisation de l’Architecture des Plantes), Boulevard de la Lironde, Montpellier, France; 4 UMR Ecolab, Université de Toulouse Paul Sabatier, CNRS, INP-ENSAT, Toulouse, France; 5 UMR EDB, CNRS, ENFA, Université de Toulouse Paul Sabatier, Toulouse, France; Chinese Academy of Medical Sciences and Peking Union Medical College, CHINA

## Abstract

The mosquito family (Diptera: Culicidae) constitutes the most medically important group of arthropods because certain species are vectors of human pathogens. In some parts of the world, the diversity is so high that the accurate delimitation and/or identification of species is challenging. A DNA-based identification system for all animals has been proposed, the so-called DNA barcoding approach. In this study, our objectives were (i) to establish DNA barcode libraries for the mosquitoes of French Guiana based on the COI and the 16S markers, (ii) to compare distance-based and tree-based methods of species delimitation to traditional taxonomy, and (iii) to evaluate the accuracy of each marker in identifying specimens. A total of 266 specimens belonging to 75 morphologically identified species or morphospecies were analyzed allowing us to delimit 86 DNA clusters with only 21 of them already present in the BOLD database. We thus provide a substantial contribution to the global mosquito barcoding initiative. Our results confirm that DNA barcodes can be successfully used to delimit and identify mosquito species with only a few cases where the marker could not distinguish closely related species. Our results also validate the presence of new species identified based on morphology, plus potential cases of cryptic species. We found that both COI and 16S markers performed very well, with successful identifications at the species level of up to 98% for COI and 97% for 16S when compared to traditional taxonomy. This shows great potential for the use of metabarcoding for vector monitoring and eco-epidemiological studies.

## Introduction

The mosquito family (Diptera: Culicidae) is composed of 3,552 valid species distributed throughout most types of ecosystems worldwide [1]. It also constitutes the most medically important group of arthropods because certain species are vectors of human pathogens, causing major health issues in some parts of the world [2]. In French Guiana, a French overseas region (84,000 km^2^) situated in South America, mosquito-borne diseases are frequent. Malaria is transmitted by *Anopheles* species mainly in inland areas of the territory [3], whereas Dengue, Chikungunya and Zika are transmitted by *Aedes* (*Stegomyia*) *aegypti* in urban areas [4; 5; 6]. Furthermore, many lesser known crypto-arboviroses occur in rural and/or sylvan environments [7]. Because these pathogens are often transmitted by a small number of vector species, their precise taxonomic identification is of primary importance to medical entomology.

French Guiana harbors one of the highest relative species densities of mosquitoes anywhere in the world [8; 9]. A recent revision of the mosquitoes of French Guiana established that 235 species have been found in the territory to date [10]. However, identification based on morphological characteristics can be challenging, especially when basic descriptive references are obsolete and/or incomplete. Even when a complete description is available, morphological identification also entails several operational hurdles. For many species, only adults have been studied, which can prevent the identification of immature stages if the mosquitoes are not reared in the laboratory. Also, morphological identification is often reliable only when the adults are in perfect condition, which is rarely the case with field-caught specimens subjected to natural and/or sampling-induced damages.

Hebert and colleagues proposed using the mitochondrial gene cytochrome *c* oxidase subunit I (COI) as a DNA-based identification system for all animal species, the so-called DNA barcoding approach [11]. Despite the limitations of the method [12], COI barcoding has proven to be particularly reliable in delimiting species for many groups of organisms like ants [13], birds [14] or fishes [15]. For mosquitoes, the suitability of the COI gene for species identification was first tested on 37 species occurring in Canada [16]. Since then, barcoding has been used for mosquito species in many parts of the world, including India [17], Iran [18], China [19], Argentina [20], Ecuador [21; 22], Pakistan [23], Singapore [24], Belgium [25], Colombia [26] and Brazil [27]. In most cases, these studies show a high correspondence between morphological species delimitation and mtDNA barcode clusters, but others point out the inability of the method to separate some closely related species distinguished by traditional taxonomy [20].

More recently, high-throughput sequencing has extended the use of DNA barcoding to the identification of multiple species from a single sample [28]. This approach, referred to as metabarcoding, allows the simultaneous identification of multiple specimens from a single bulk-DNA extraction [29; 30]. While the COI marker has been used as a standard in barcoding applications, it is not the best choice when it comes to metabarcoding [31] and a shorter fragment in the 16S ribosomal gene has been specifically designed for metabarcoding applications for insects [32]. It was recently successfully used to analyze samples of Phlebotomine sandflies [30].

In this study, our objectives were three-fold: (i) to establish DNA barcode libraries for the mosquito fauna of French Guiana based on the COI and the 16S markers, (ii) to compare distance-based and tree-based methods of species delimitation to traditional taxonomy, and (iii) to evaluate the accuracy of each marker in identifying specimens.

## Materials and methods

### Ethics statement

This study was conducted according to the relevant national and international guidelines and did not involve endangered or protected species. Mosquito sampling was authorized by the French *Office National des Forêts* (ONF). Specific sampling authorizations were also obtained from the *Réserve Naturelle Nationale* managed by the ONF for the Montagnes de la Trinité, and from the *Parc Amazonien de Guyane* (PAG) for the field mission conducted at Mont Itoupé. Note that sampling carried out on private land was always conducted after receiving the permission from the owner.

### Sampling and a priori identification

Sampling was conducted in various habitats in French Guiana between 2013 and 2015 [33; 34]. The following locations were sampled: Cayenne (4.913°N, 52.303°W), Kourou (5.168°N, 52.642°W), Macouria (5.014°N, 52.474°W), Matoury (4.851°N, 52.331°W), Mont Itoupé (3.023°N, 53.084°W), Montagnes de la Trinité (4.583°N, 53.343°W), Montsinéry (4.893°N, 52.493°W), Petit-Saut (5.066°N, 53.050°W), Régina (4.314°N, 52.129°W), Roura (4.728°N, 52.324°W), Saül (3.623°N, 53.210°W) and Sinnamary (5.377°N, 52.958°W). Immature container-inhabiting mosquitoes were collected by extracting water using a great variety of sucking devices in order to fit the variety of structures and water volumes. On several occasions, natural and artificial ovitraps were used, including bamboo stumps and artificial bromeliads installed at ground or canopy level. Immature mosquitoes from larger bodies of water were collected using a kick net. Adult mosquitoes were attracted in the field by human bait and captured using a butterfly net or, if settled, a tube. All of the samples used in this study were integrated into an online database record [33] available through the Global Biodiversity Information Facility (GBIF) data portal at http://www.gbif.org/dataset/5a8aa2ad-261c-4f61-a98e-26dd752fe1c5/ or through the Guyanensis platform (http://guyanensis.ups-tlse.fr/).

Whenever possible, samples were brought back alive to the laboratory. Immature mosquitoes were individually reared in 2 mL tubes and placed in an environmental chamber at 28°C in order to obtain adults. Fourth instar and pupal skins were sorted and stored in individual tubes containing 70% ethanol. When a sufficient number of adults was obtained, immatures were killed and stored in individual tubes containing 96% ethanol. Reared adults and those captured in the field were freeze-killed. Three legs from the right lateral side of each specimen were then carefully dissected on ice and kept in a separate vial containing 96% ethanol and stored at -20°C for further molecular investigations. Adults were mounted on their right side on a pin point and stored in entomological boxes. Specimen codes are based on the name of the collection followed by a unique serial number as proposed by Gaffigan and Pecor [35]. The same code was used for all of the biological material issued from the same specimen. When it was not possible to bring live samples back to the laboratory or to rear them, specimens were stored directly in the field in 96% ethanol. The identifications of specimens were made by the first author, often based on the examination of both immature and adult specimens, and by using the latest publications on the genus or on the subgenus concerned (see [10]). Most of the specimens sampled were identified to species level and, when this was not possible, we created classifications of morphospecies using the genus name followed by the suffix ‘sp.st’ associated with a capital letter.

### Sequencing

DNA was extracted from two legs of each adult specimen or from a larval head (S1 Table) using the DNeasy Blood and Tissue Kit (Qiagen, Valencia, CA, USA). The standard 658 base pairs barcode of the mitochondrial Cytochrome *c* Oxidase subunit I gene (COI) was amplified using the primers LCO1490/HCO2198 [36]. The total PCR volume was 25 *μ*L and consisted of 2.5 *μ*L of 10X reaction buffer, 2 *μ*L of 2.5 mM dNTPs, 2 *μ*L of 25 mM MgCl_2_, 0.5 *μ*L of each 10 *μ*M primer, 0.2 *μ*L of 5U/L Taq Polymerase, 15.3 *μ*L of H_2_O and 2 *μ*L of template DNA. The PCR cycles were as follows: 94°C for 2 min, 40 cycles at 94°C for 30 s, 49°C for 45 s and 72°C for 45 s, and then a final extension at 72°C for 1 min. The ‘insect metabarcode’ marker was amplified using the Ins16S_1 primer pair ([32]; Ins16S_1-F 5’- TRRGACGAGAAGACCCTATA-3’; Ins16S_1-R 5’- TCTTAATCCAACATCGAGGTC-3’). The total PCR volume was 26.8 *μ*L and consisted of 2.7 *μ*L of 10X reaction buffer, 1.7 *μ*L of 2 mM dNTPs, 2.7 *μ*L of 50 mM MgCl_2_, 1.3 *μ*L of each 10 *μ*M primer, 0.3 *μ*L of 5U/L Taq Polymerase, 10 *μ*L of H_2_O and 6.8 *μ*L of template DNA. The PCR cycles were as follows: 95°C for 5 min, 35 cycles at 94°C for 30 s, 50°C for 30 s and 72°C for 30 s, and then a final extension at 72°C for 7 min. The PCR products for each marker were verified on 2% agarose gel and were commercially sequenced on an ABI3730 by Genoscreen (Lille, France). Forward and reverse sequences were edited and assembled using Geneious 9 (http://www.geneious.com/; [37]). All sequences were uploaded to the Barcode of Life Data Systems (BOLD; [38]) and can be found under BOLD accession numbers FGMOS001-16 to FGMOS1244-16.

### Species delimitation

Several algorithms for molecular species delimitation exist. They can be broadly classified into two categories: distance-based methods and phylogeny-based methods. We took into consideration two implementations that do not rely on *ad hoc* similarity thresholds and do not require parameters that are difficult to select *a priori*.

As a distance-based method, we used the REfin Single Linkage clustering approach (RESL; [39]) to define Barcode Index Numbers (BINs) based on our COI dataset. The RESL algorithm has the advantage of using a two-step procedure: an initial clustering at a 2.2% divergence threshold followed by a refinement step using Markov clustering. In addition, it uses all of the sequences present in the BOLD database for clustering, allowing for a direct comparison of our dataset with sequences produced from other barcoding projects such as ACMC (Mosquitoes of North America), CULBE (DNA barcoding of Belgian mosquito species), MEA (Mosquitoes of the Ecuadorian Amazon) or mined from Genbank (BBDCU).

As a tree-based method, we used the Poisson Tree Process [40] as implemented in mPTP [41]. The method seeks to classify the branches of a phylogenetic tree into two processes: within species (corresponding to a coalescence process) and between species (corresponding to a speciation process). Because the method uses a phylogenetic tree, we first performed a phylogenetic analysis of our dataset by combining the COI and the 16S data and performing a Maximum Likelihood analysis in RAxML v8 [42], applying a GTR+ Gamma model to each partition and an automatic bootstrapping procedure to assess nodal support. Delimitation support values were inferred using a Markov Chain Monte Carlo sampling approach, using five independent runs of 10 million steps and discarding the first two million as burning.

### Specimen identification

Distance measures of identification success were computed based on the pairwise Kimura 2-Parameter distance matrix of the multiple sequence alignment for each marker using the R package Spider [43]. We first used the ‘nearest-neighbour’ criterion (also known as ‘best match’), which simply finds the closest individual to the query and return the species for that individual as identification for the query. In the case of an incomplete reference library, the rate of false-positives can be high as query sequences will always be assigned to a matching sequence regardless of its distance (i.e. species not present in the database will be assigned to the closed species even though it is highly dissimilar). The ‘best close match’ is another distance-based criterion that incorporates a threshold in order to circumvent the problem of the ‘best match’ criterion [44]. Any sequence above a certain threshold (i.e. potentially species not present in the database) will not be assigned. When multiple equally close matches are retrieved, the assignation can be correct (all matches are the same species), incorrect (all matches are species different from the query) or ambiguous (both correct and incorrect matches are retrieved). Finally, the ‘BOLD ID’ criterion (also known as ‘threshID’ or ‘all species barcode’) operates on all matches within the threshold rather than the ‘nearest-neighbour’ match as in the ‘best close match’ criterion. For all of the analyses, we optimized the threshold value by minimizing the false positive (no conspecific matches within query threshold) and false negative (non-conspecific species within the threshold distance of query). For all of the analyses, singletons (species represented by only one individual) were removed from the results. However, those specimens were kept in the analyses and are still available as potential mismatches for other species. All of the analyses were performed using either traditional taxonomy (species as they are delimited by morphological analysis) or molecular species (as defined by the BINs). For the 16S dataset, we removed the sequences that were not complete; usually, these were a few base pairs at the 5’end due to the low quality of the reverse read.

In order to further evaluate the reliability of the 16S maker in the context of metabarcoding, we used the ecotag program [45], which is now widely used for the taxonomic assignation of metabarcoding reads (e.g. [30]). Because of the short length of the sequences, genetic distances are computed based on pairwise alignments rather than on the multiple sequence alignment of all sequences. In addition, it uses raw distances based on the longest common subsequences rather than corrected distances. Finally, uncertainty is taken into account using the ‘last common ancestor’ algorithm. The program ecotag first searches for the reference sequence(s) showing the highest similarity with the query sequence (primary reference sequence(s)). Then it looks for all other reference sequences whose similarity with the primary reference sequence(s) is equal or higher than the similarity between the primary reference sequence(s) and the query sequence (secondary reference sequence(s)). Finally, it assigns the query sequence to the most recent common ancestor of the primary and secondary reference sequences.

## Results

### Species delimitation

**Fig 1:**
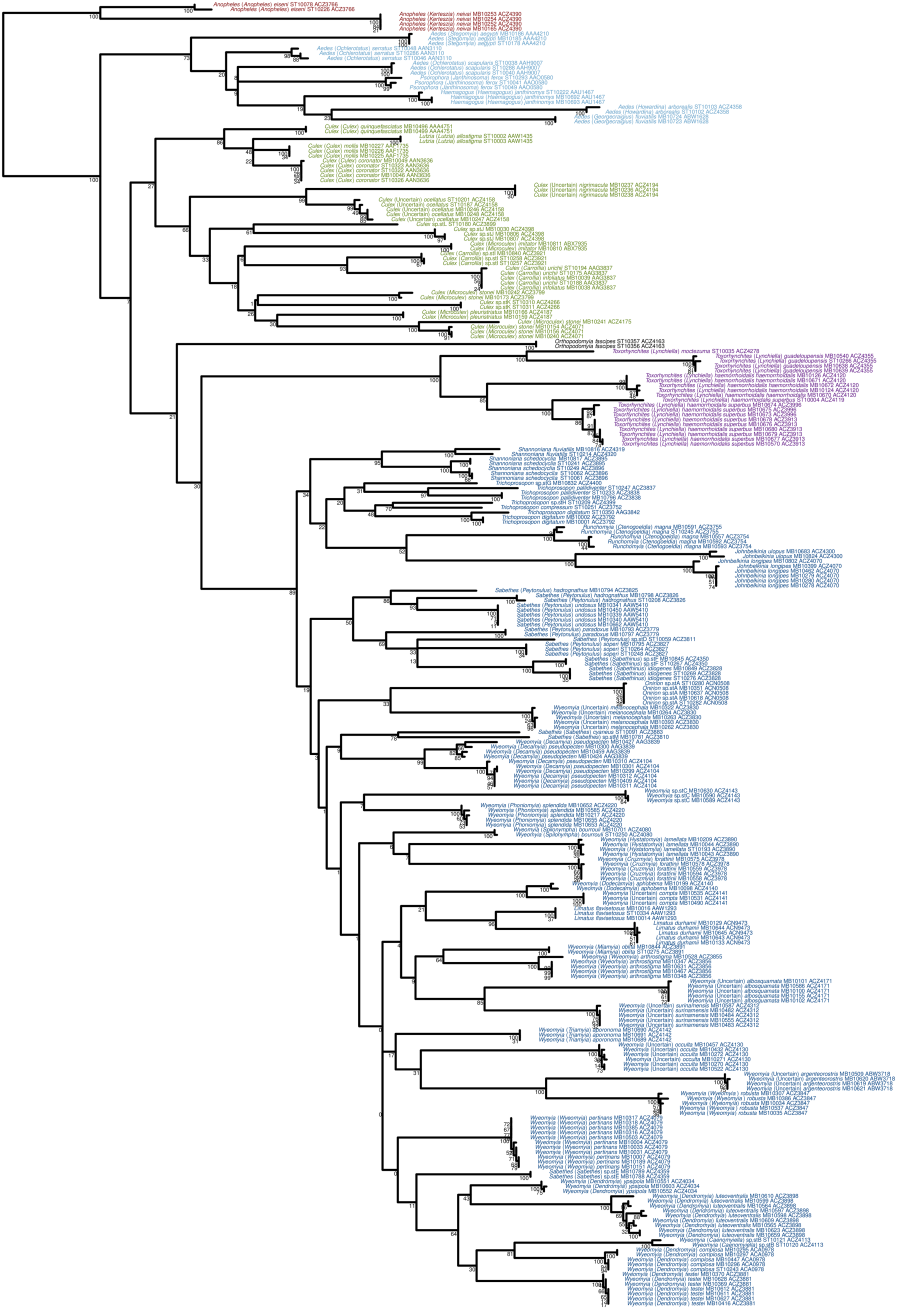
Maximum Likelihood (ML) phylogenetic analysis of the combined COI and 16S dataset. Bootstrap support values are indicated below the branches. For each specimen, we indicated the traditional taxonomic identification, the specimen code and the Barcode Index Number (BIN). The main taxonomic groups are colour-coded: Anophelinae in red, among the Culicinae, Aedini in turquoise, Culicini in green, Orthopodomyiini in grey, Sabethini in blue and Toxorhynchitini in purple.

A total of 266 morphologically identified specimens belonging to 75 species or morphospecies grouped within 16 genera were analyzed (S1 Table). The RESL clustering approach applied to the COI marker allowed us to distinguish 86 BINs (S2 Table). The results of the clustering approach were largely congruent with the morphological delimitations (Fig 1). We found one case where two nominal species (namely, *Cx*. (*Car*.) *infoliatus* and *Cx*. (*Car*.) *urichii*) were clustered into a single BIN (AAG3837). In 10 cases, nominal species were split into one or more BINs; namely: *Cx*. (*Mcx*.) *stonei* (BINs ACZ3799, ACZ4071 and ACZ4175), *Ru*. (*Cte*.) *magna* (BINs ACZ3754 and ACZ3755), *Sa*. (*Pey*.) *hadrognathus* (BINs ACZ3825 and ACZ3826), *Sh*. *fluviatilis* (BINs ACZ4319 and ACZ4320), *Sh*. *schedocyclia* (BINs ACZ3895 and ACZ3896), *Tr*. *digitatum* (BINs AAG3842 and ACZ3792), *Tr*. *pallidiventer* (BINs ACZ3837 and ACZ3838), *Wy*. (*Dec*.) *pseudopecten* (BINs AAG3839 and ACZ4104), *Wy*. (*Wyo*.) *arthrostigma* (BINs ACZ3855 and ACZ3856) and *Tx*. (*Lyn*.) *haemorrhoidalis superbus* (BINs ACZ3913, ACZ3996 and ACZ4119).

Among the 86 BINs present in our dataset, 21 BINs include sequences already present in BOLD. We observed 12 cases of perfect clustering: *Ae*. (*Gec*.) *fluviatilis* (BIN ABW1628); *Ae*. (*Och*.) *scapularis* (BIN AAH9007); *Ae*. (*Och*.) *serratus* (BIN AAN3110); *Ae*. (*Stg*.) *aegypti* (BIN AAA4210, despite a few BOLD specimens that might have been misidentified); *Hg*. (*Hag*.) *janthinomys* (BIN AAU1467); *Ps*. (*Jan*.) *ferox* (BIN AAO0580); *Cx*. (*Mcx*.) *imitator* (BIN ABX7935); *Lt*. (*Lut*.) *allostigma* (BIN AAW1435); *Li*. *durhamii* (BIN ACN9473); *Li*. *flavisetosus* (BIN AAW1293); and *Wy*. (*Den*.) *complosa* (BIN ACA0978).

In five cases, there was a mismatch between our identifications and the ones present in the other datasets: BIN AAG3837 included *Cx*. (*Car*.) *infoliatus* and *Cx*. (*Car*.) *urichii* and clusters with *Cx*. (*Car*.) *urichii* (9 counts); BIN AAN3636 identified as *Cx*. (*Cux*.) *coronator* clusters with *Cx*. (*Cux*.) *maxi* Dyar 1928 (76 counts), *Cx*. (*Cux*.) *coronator* (21 counts) and other identified/unidentified *Culex* species (26 counts); BIN AAF1735 identified as *Cx*. (*Cux*.) *mollis* clusters with *Cx*. (*Cux*.) *nigripalpus* Theobald 1901 (64 counts), *Cx*. (*Cux*.) *interfor* Dyar 1928 (43 counts) and several other identified/unidentified *Culex* species (80 counts); and BIN AAA4751 identified as *Cx*. (*Cux*.) *quinquefasciatus* clusters with *Cx*. (*Cux*.) *quinquefasciatus* (1971 counts) and *Cx*. (*Cux*.) *pipiens* s.l. Linnaeus 1758 (1186 counts); and BIN ACZ4079 identified as *Wy*. (*Wyo*.) *pertinans* clusters with one specimen of *Wy*. (*Wyo*.) *mitchellii* (Theobald 1905) from Venezuela.

In five other cases, the BINs clustered with only unidentified specimens in BOLD: *Onirion* sp.stA (BIN ACN0508), *Sa*. (*Pey*.) *undosus* (BIN AAW5410), *Tr*. *digitatum* (BIN AAG3842), *Wy*. (Uncertain) *argenteorostris* (BIN ABW3718) and *Wy*. (*Dec*.) *pseudopecten* (BIN AAG3839).

The PTP method was largely congruent with the distance-based approach and resulted in the definition of 87 MOTUs (*vs* 86 for RESL) with minor differences. The specimen MB10610 (*Wy*. (*Den*.) *luteoventralis*) was separated from BIN ACZ3898 and specimens of BIN ACZ3766 (*An*. (*Ano*.) *eiseni*) were separated into two MOTUs. Contrarily, ACZ3996 and ACZ3913 (both belonging to *Tx*. (*Lyn*.) *haemorrhoidalis superbus*) were grouped together within the same MOTU.

### Specimen identification

Of the 266 specimens available, eight species (considered at the traditional taxonomy level) were represented by only one specimen and thus could not be included in our identification test. The final statistics were thus calculated using a total of 259 specimens. When placed at the BIN level, 18 BINs were represented by only one specimen and the final statistics were based on 249 specimens. When using the nearest-neighbour method, we found the COI marker to be accurate to 98% at the species level and 100% at the BIN level (Table 1). This is because of the five specimens of *Cx*. (*Car*.) *urichii* and *Cx*. (*Car*.) *infoliatus* that are grouped within a single BIN. When using ‘best close match’ and ‘BOLD ID’, the rates are of 95.8% and 98.7% because few specimens result in ‘no ID’ results (Table 1). At the BIN level, these were MB10802 *Jb*. *longipes*, MB10427 *Wy*. (*Dec*.) *pseudopecten* and MB10610 *Wy*. (*Den*.) *luteoventralis*, which were above the threshold of identification success but below the threshold for BIN delimitation.

**Table 1:** Identification success using the Kimura-2 parameter distances with three different criteria: ‘Nearest-neighbour’, ‘best close match’ and ‘BOLD ID’.

Criterion	Success rate	Correct	Ambiguous	Incorrect	No ID	Threshold
**COI (species level)**						
Nearest-neighbour	98%	254		5		
Best close match	95.8%	248	5	0	6	0.025
BOLD ID	95.8%	248	5	0	6	0.025
**COI (BIN level)**						
Nearest-neighbour	100%	249		0		
Best close match	98.7%	246	0	0	3	0.013
BOLD ID	98.7%	246	0	0	3	0.013
**16S (species level)**						
Nearest-neighbour	97%	195		6		
Best close match	94%	189	5	0	7	0.019
BOLD ID	85.1%	171	23	0	7	0.019
**16S (BIN level)**						
Nearest-neighbour	97.4%	185		5		
Best close match	86.8%	165	11	1	13	0.010
BOLD ID	74.7%	142	35	0	13	0.010

For the 16S marker, we removed the specimens for which the sequences were shorter than expected due to low quality of the reverse reads. The final dataset was thus composed of 211 sequences with 201 sequences for the statistics at the species level and 190 at the BIN level. We found an identification success of 97% (species level) and 97.4% (BIN level) using the ‘best match’ criterion (Table 1). This is again related to the *Cx*. (*Car*.) *urichii* / *Cx*. (*Car*.) *infoliatus* case, plus MB10794 (*Sa*. (*Pey*.) *hadrognathus*) which is highly dissimilar to the remaining specimens of *Sa*. (*Pey*.) *hadrognathus*. Using the ‘best close match’ criterion, we found one incorrect assignation at the BIN level for MB10592 (*Ru*. (*Cte*.) *magna*), which was assigned to another BIN from the same species. When using the ‘last common ancestor’ approach as implemented in ecotag, we found that 100% of the sequences were correctly assigned, with 97% assigned to the species level, five sequences assigned to the genus level and only one sequence assigned to the tribe level.

## Discussion

In the present study, we have assessed and compared the usefulness of barcode and metabarcode markers in delimiting and identifying poorly known Neotropical culicid species. Overall, based on a dataset of 75 morphologically identified species, we obtained 11% more taxa using molecular delimitation than with morphology-based identification. This difference might be due to three factors: the presence of complexes of closely related species (i.e. cryptic species), the high sequence divergence of some species and the gap in basic taxonomic knowledge. We discuss below which is the most likely hypothesis for each taxa split into more than one BIN.

*Culex* (*Mcx*.) *stonei* specimens (MB10154, 0156, 0173, 0240, 024, 0242) clustered in three different BINs. This result is unexpected because the specimens were collected on the same date and from the same location which might suggest the presence of cryptic species occurring in sympatry, or that the high sequence divergence within this species is not adequately represented in our sampling.

*Sabethes* (*Pey*.) *hadrognathus* was described by Harbach as part of the thorough revision of the subgenus which began in 1991 [46; 47; 48; 49; 50]. MB10794, 0798 and STI0208 constitute the three sole specimens of *Sa*. (*Pey*.) *hadrognathus* ever caught in French Guiana [33]. The molecular delimitation of *Sa*. (*Pey*.) *hadrognathus* into two BINs suggests the presence of two closely related species which might be one of the three species of *Peytonulus* whose larval stage is unknown (i.e. *Sa*. (*Pey*.) *gorgasi* Duret 1971, *Sa*. (*Pey*.) *ignotus* Harbach 1995 or *Sa*. (*Pey*.) *xenismus* Harbach 1995) or one of the undescribed species [50]. Further examination of additional specimens at all life stages will be needed to determine if morphological characteristics support the presence of another species or simply that intraspecific divergence within this taxon is high.

All of the nominal species *Ru*. (*Cte*.) *magna*, *Sh*. *fluviatilis*, *Sh*. *schedocyclia*, *Tr*. *digitatum* and *Tr*. *pallidiventer* were split into two BINs. These species belong to the same taxonomic group (formerly *Trichoprosopon sensu* Lane and Cerqueira [51]) which was the subject of a key revision by Zavortink in 1979. In this revision, Zavortink pointed out the difficulties in identifying the different species belonging to the genera *Runchomyia*, *Shannoniana* and *Trichoprosopon* given that most of the available descriptions are insufficient and/or incomplete [52]. The situation has not evolved since the Seventies and our results probably reflect the lack of precise and complete descriptions of species.

*Wyeomyia* (*Dec*.) *pseudopecten* was also split into two BINs. The three species currently included in the subgenus *Decamyia* have not been studied in detail, particularly immatures [1]. For example, at the larval stage, *Wy*. (*Dec*.) *pseudopecten* and *Wy*. (*Dec*.) *ulocoma* (Theobald 1903) cannot be unfailingly distinguished. The two sequenced males (MB10424, 0427) belonged to the same BIN and definitely harbored characters of *Wy*. (*Dec*.) *pseudopecten* that belongs to a group of species including *Wy*. (*Dec*.) *ulocoma* (Theobald 1903), *Wy*. (*Dec*.) *felicia* (Dyar & Núñez Tovar 1927) and probably *Wy*. (Uncertain) *rorotai* Senevet, Chabelard & Abonnenc 1942. Like many other infra-generic subgroups within the genus *Wyeomyia*, the *Decamyia* subgenus of *Wyeomyia* deserves a thorough revision [53].

It is likely that *Tx*. (*Lyn*.) *haemorrhoidalis superbus* constitutes a complex of closely related species because the specimens were split into three BINs. Two BINs grouped specimens based on their sampling site: Cayenne (MB10673, 0674, 0675) or Régina and Petit-Saut (MB10570, 0676, 0677, 0678, 0679, 0680). The third BIN corresponded to one individual (ST10004) collected in the deep primary forest of Petit-Saut; this fact is unusual as all other specimens were collected along forest edges.

In a few cases, there was a mismatch between our identifications and the one present in BOLD. For example, our specimens identified as *Wy*. (*Wyo*.) *pertinans* clustered with one specimen collected in Venezuela and identified as *Wy*. (*Wyo*.) *mitchellii*. Both species belong to the Pertinans group of *Wyeomyia* which includes at least 13 closely related species distributed across the Americas and records of *Wy*. (*Wyo*.) *mitchellii* in Central and South America are erroneous[54]. As a consequence, this record should be interpreted as a misidentification.

The Coronator complex of *Culex* comprises six species distributed across the Americas and only separated on the basis of male genitalia and distribution [55]. Our specimens of *Cx*. (*Cux*.) *coronator* (MB10046, 0049 and ST10322, 0323, 0326) clustered with *Cx*. (*Cux*.) *maxi*, *Cx*. (*Cux*.) *coronator* and other *Culex* species belonging or not to the Coronator complex. Because the specimens were identified based on the structure of the apical lobe of the basistyle of the male genitalia [55], we are quite confident of our identification. In addition, our specimens identified as *Cx*. (*Cux*.) *mollis* (MB10225, 0226, 0227) clustered with *Cx*. (*Cux*.) *nigripalpus*, *Cx*. (*Cux*.) *interfor* and other *Culex* species in BOLD. Morphological identifications in this case were only based on the larval stage, yet the differences at this stage are slight between these species so that our identification is questionable. Also, *Cx*. (*Cux*.) *quinquefasciatus* clustered with *Cx*. (*Cux*.) *quinquefasciatus* as well as with *Cx*. (*Cux*.) *pipiens* s.l., its temperate equivalent [56]. As already pointed out by other authors, our results confirm that the COI barcode does not contain enough information to distinguish closely related species among the subgenus *Culex* [20]).

All of the morphospecies included in the analyses (namely, sp.stA to sp.stM) have been confirmed to be distinct from other related taxa and did not match any identified species in BOLD. Potentially, each of them represents an undescribed species or, at least, an undescribed life stage of an incompletely described species. Because most of them are represented by very few specimens, further field missions will be necessary to gather enough biological material to allow their precise and complete description.

The phylogenetic analysis of the combined dataset (COI + 16S) was originally designed to perform a tree-based species delimitation approach which proved to be highly congruent with the distance-based species delimitation. Even though it was not the aim of this study, the resulting topology offers the opportunity to discuss some phylogenetic aspects. We found that all of the tribes present are monophyletic and supported by high bootstrap values with the exception of the tribe Culicini. However, many of the genera were not found to be monophyletic and/or weakly supported by bootstrap values. Additional markers should be used in the future to resolve the intra- and inter-generic relationships as well as the deeper nodes at inter-tribal level. Nevertheless, some of the species formed clusters that are worth noting. Among the tribe Culicini, *Cx*. *nigrimacula* and *Cx*. *ocellatus* clustered together with a high bootstrap value (99%). These two species are among the very few *Culex* species without subgeneric placement (7/770 species; [1]). This result validates their common evolutionary relationship which is strongly corroborated by morphological characteristics at all life stages. *Lutzia allostigma* and the three species of the subgenus *Culex* included in the analysis clustered in a well supported clade with a bootstrap value of 86%. This result confirms the affinities between *Lutzia* and the subgenus *Culex* as stated by Belkin [57; 58] and through a molecular phylogeny based on the ITS1 and ITS2 rDNA markers [59]. More recently, *Lutzia* has been elevated to genus without having undergone any specific analysis [60]. However, because the position of *Lutzia* is not well defined in our analysis, we are unable to have an opinion on the taxonomic rank of this genus. Also, among the tribe Sabethini, six species of the genus *Wyeomyia* clustered in pairs including one or two species without subgeneric placement [53]. *Wyeomyia albosquamata* clustered with *Wy*. *surinamensis* with a high bootstrap value (85%) and both pairs composed of *Wy*. (*Dod*.) *aphobema* / *Wy*. *compta* and *Wy*. *argenteorostris* / *Wy*. (*Wyo*.) *robusta* clustered with very high bootstrap values (100%). These results indicate that *Wy*. *compta* and *Wy*. *argenteorostris* should be placed within the subgenera *Dodecamyia* and *Wyeomyia*, respectively. Moreover, *Wy*. (*Den*.) *complosa* clustered with *Wy*. (*Cae*.) sp.stB (81% bootstrap value) which is in keeping with the affinities between the two subgenera already proposed based on morphological characteristics only [53].

Finally, our results confirm that the COI barcode can be successfully used for delimiting and identifying mosquito species, with only a few cases where the marker could not distinguish closely related species. When compared to the BIN level, the COI marker has a success rate of 100%, which is expected since the BINs are defined based on the COI marker. We suggest using traditional taxonomy as a reference until further specimens are included and their comparison using morphology is thoroughly assessed. In addition, we also suggest using the ‘best close match’ rather than the ‘nearest-neighbour’ criterion because specimen identification in the Neotropics and especially in the Amazonian region is unlikely to be performed with exhaustive reference databases. Based on these recommendations, the success rate of the COI and 16S markers is 95.8% and 94%, respectively. Most notably, none of the marker gave incorrect results. When using ecotag, 100% of the assignations for the 16S were correct with 97% made at the species level. This also suggests that despite its small size (216 bp *vs* 658 bp), the 16S ‘insect metabarcode’ marker had an identification success rate similar to the classical COI barcode, opening up great opportunities for the use of metabarcoding for vector monitoring and eco-epidemiological studies.

## Conclusions

Our analysis of 266 mosquito specimens belonging to 75 morphologically identified species from French Guiana resulted in the definition of 86 DNA clusters (BINs) with only 21 BINs already present in the BOLD database, thus providing a substantial contribution to the global mosquito barcoding initiative. We confirm the presence of several new species identified based on their morphology plus several potential cases of cryptic species. Our results also confirm that DNA barcoding can be successfully used for delimiting Neotropical mosquito species as congruent results were obtained using distance-based and tree-based methods with only a few cases where the marker could not distinguish closely related species. In addition, the identification success rates of the COI and 16S markers were sensibly similar, suggesting that the metabarcoding of bulk samples of mosquitoes can be performed using the 16S ‘insect metabarcode’ marker with great accuracy. While our study was primarily designed for container-inhabiting mosquito species, our conclusions on the utility of the COI and 16S markers should be applied to a broader range of mosquitoes including ground pool-inhabiting species.

## Supporting information

S1 Table List of the mosquito species or morphospecies (hereafter ‘taxa’) corresponding to the voucher specimens that were COI and 16S sequenced in this study. Taxa are listed alphabetically and ranked by subfamily, tribe, genus and subgenus. The life stage is indicated for each taxa (M: male; F: female; L: larva).
(DOCX)

S2 Table List of Barcode Index Numbers (BINs) with their associated species or morphospecies (hereafter ‘taxa’) obtained from the Barcode of Life Data Systems (BOLD; last visited august 2016). Taxa are listed alphabetically and ranked by subfamily, tribe, genus and subgenus. Distances (p-distance) correspond to the percentage of dissimilar pairwise nucleotides and counts correspond to the number of voucher specimens included in this study followed, between brackets, by the total number of specimens (including ours) present in the BOLD database.
(DOCX)
