# Supplementary material for: DNA reference libraries of French Guianese mosquitoes for barcoding and metabarcoding
Source: PLoS One. 2017 Jun 2;12(6):e0176993. doi: 10.1371/journal.pone.0176993 (PMC5456030; doi:10.1371/journal.pone.0176993)
Supplement: S1 Table — Taxa are listed alphabetically and ranked by subfamily, tribe, genus and subgenus. The life stage is indicated for each taxa (M: male; F: female; L: larva). (DOCX) [file pone.0176993.s001.docx]

**S1 Table. List of the mosquito species or morphospecies (hereafter ‘taxa’) corresponding to the voucher specimens that were COI and 16S sequenced in this study. Taxa are listed alphabetically and ranked by subfamily, tribe, genus and subgenus. The life stage is indicated for each taxa (M: male; F: female; L: larva).**

| **Species/morphospecies** | **Voucher specimen** | **Life stage** |
| --- | --- | --- |
| **Anophelinae** |  |  |
| *Anopheles* (*Anopheles*) *eiseni* Coquillett 1902 | ST10078, ST10226 | M/L |
| *Anopheles* (*Kerteszia*) *neivai* Howard, Dyar & Knab 1913 | MB10165, MB10252, MB10253, MB10254 | F/L |
| **Culicinae: Aedini** |  |  |
| *Aedes* (*Georgecraigius*) *fluviatilis* (Lutz 1904) | MB10723, MB10724 | L |
| *Aedes* (*Ochlerotatus*) *scapularis* (Rondani 1848) | ST10038, ST10040, ST10288 | F/L |
| *Aedes* (*Ochlerotatus*) *serratus* (Theobald 1901) | ST10046, ST10048, ST10286 | F/L |
| *Aedes* (*Howardina*) *arborealis* Bonne-Wepster & Bonne 1920 | ST10102, ST10103 | L |
| *Aedes* (*Stegomyia*) *aegypti* (Linnaeus 1762) | ST10178, MB10185, MB10186 | M/F/L |
| *Haemagogus* (*Haemagogus*) *janthinomys* Dyar 1921 | MB10692, MB10693, ST10222 | F/L |
| *Psorophora* (*Janthinosoma*) *ferox* (von Humboldt 1819) | ST10041, ST10049, ST10293 | F/L |
| **Culicinae: Culicini** |  |  |
| *Culex* (*Carrollia*) *infoliatus* Bonne-Wepster & Bonne 1920 | MB10038, MB10039 | M/F |
| *Culex* (*Carrollia*) *urichii* (Coquillett 1906) | ST10175, ST10188, ST10194 | M/F/L |
| *Culex* (*Carrollia*) sp.stI | MB10840, ST10257, ST10258 | M/F/L |
| *Culex* (*Culex*) *coronator* Dyar & Knab 1906 | MB10046, MB10049, ST10322, ST10323, ST10326 | M/F/L |
| *Culex* (*Culex*) *mollis* Dyar & Knab 1906 | MB10225, MB10226, MB10227 | L |
| *Culex* (*Culex*) *quinquefasciatus* Say 1823 | MB10496, MB10499 | M/F |
| *Culex* (*Microculex*) *imitator* Theobald 1903 | MB10810, MB10811 | L |
| *Culex* (*Microculex*) *pleuristriatus* Theobald 1903 | MB10159, MB10166 | M/F |
| *Culex* (*Microculex*) *stonei* Lane & Whitman 1943 | MB10154, MB10156, MB10173, MB10240, MB10241, MB10242 | M/F/L |
| *Culex* (Uncertain) *nigrimacula* Lane & Whitman 1943 | MB10236, MB10237, MB10238 | L |
| *Culex* (Uncertain) *ocellatus* Theobald 1903 | MB10246, MB10247, MB10248, ST10187, ST10201 | M/F/L |
| *Culex* sp.stJ | MB10030, MB10806, MB10807 | F/L |
| *Culex* sp.stK | ST10310, ST10311 | L |
| *Culex* sp.stL | ST10180 | F |
| *Lutzia* (*Lutzia*) *allostigma* Dyar & Knab 1915 | ST10002, ST10003 | M/F |
| **Culininae: Orthopodomyiini** |  |  |
| *Orthopodomyia fascipes* (Coquillett 1906) | ST10356, ST10357 | L |
| **Culicinae: Sabethini** |  |  |
| *Johnbelkinia longipes* (Fabricius 1805) | MB10278, MB10279, MB10280, MB10399, MB10462, MB10802 | M/F/L |
| *Johnbelkinia ulopus* (Dyar & Knab 1906) | MB10683, MB10802 | M/F/L |
| *Limatus durhamii* Theobald 1901 | MB10129, MB10133, MB10643, MB10644, MB10645 | M/F/L |
| *Limatus flavisetosus* de Oliveira Castro 1935 | MB10014, MB10016, ST10334 | M/F/L |
| *Onirion* sp.stA | MB10351, MB10618, MB10637, ST10280, ST10282 | M/F/L |
| *Runchomyia* (*Ctenogoeldia*) *magna* (Theobald 1905) | MB10557, MB10591, MB10592, MB10593, ST10245 | M/F/L |
| *Sabethes* (*Peytonulus*) *hadrognathus* Harbach 1995 | MB10794, MB10798, ST10208 | M/L |
| *Sabethes* (*Peytonulus*) *paradoxus* Harbach 2002 | MB10793, MB10797 | L |
| *Sabethes* (*Peytonulus*) *soperi* Lane & Cerqueira 1942 | MB10795, ST10248, ST10264 | M/F/L |
| *Sabethes* (*Peytonulus*) *undosus* (Coquillett 1906) | MB10339, MB10340, MB10341, MB10450, MB10662 | M/F/L |
| *Sabethes* (*Peytonulus*) sp.stD | ST10059 | L |
| *Sabethes* (*Sabethes*) *cyaneus* (Fabricius 1805) | ST10091 | F |
| *Sabethes* (*Sabethes*) sp.stE | MB10788, MB10789 | L |
| *Sabethes* (*Sabethes*) sp.stM | MB10781 | L |
| *Sabethes* (*Sabethinus*) *idiogenes* Harbach 1994 | MB10849, ST10269, ST10276 | M/F/L |
| *Sabethes* (*Sabethinus*) sp.stF | MB10845, ST10267 | F/L |
| *Shanoniana fluviatilis* (Theobald 1903) | MB10816, ST10214 | M/L |
| *Shanoniana schedocyclia* (Dyar & Knab 1908) | MB10817, ST10061, ST10062, ST10241, ST10249 | M/F/L |
| *Trichoprosopon compressum* Lutz 1905 | ST10251 | M |
| *Trichoprosopon digitatum* (Rondani 1848) | MB10001, MB10002, ST10350 | M/F/L |
| *Trichoprosopon pallidiventer* (Lutz 1905) | MB10796, ST10247, ST10233 | M/F/L |
| *Trichoprosopon* sp.stG | MB10832 | L |
| *Trichoprosopon* sp.stH | ST10209 | F |
| *Wyeomyia* (*Caenomyiella*) sp.stB | ST10120, ST10121 | L |
| *Wyeomyia* (*Cruzmyia*) *forattinii* Clastrier 1974 | MB10558, MB10559, MB10575, MB10578, MB10594 | F/L |
| *Wyeomyia* (*Decamyia*) *pseudopecten* Dyar & Knab 1906 | MB10299, MB10300, MB10301, MB10310, MB10311, MB10312, MB10409, MB10424, MB10427, MB10459 | M/F/L |
| *Wyeomyia* (*Dendromyia*) *complosa* (Drar 1928) | MB10295, MB10296, MB10297, MB10447, ST10243 | M/F/L |
| *Wyeomyia* (*Dendromyia*) *luteoventralis* Theobald 1901 | MB10564, MB10565, MB10597, MB10598, MB10599, MB10609, MB10610, MB10623, MB10659 | M/F/L |
| *Wyeomyia* (*Dendromyia*) *testei* Senevet & Abonnenc 1939 | MB10370, MB10369, MB10416, MB10611, MB10612, MB10627, MB10628 | F/L |
| *Wyeomyia* (*Dendromyia*) *ypsipola* Dyar 1922 | MB10551, MB10552, MB10603 | L |
| *Wyeomyia* (*Dodecamyia*) *aphobema* Dyar 1918 | MB10098, MB10199 | L |
| *Wyeomyia* (*Hystatomyia*) *lamellata* (Bonne-Wepster & Bonne 1919) | MB10043, MB10044, MB10209, ST10193 | M/L |
| *Wyeomyia* (*Miamyia*) *oblita* (Lutz 1904) | MB10844, ST10275 | M/L |
| *Wyeomyia* (*Phoniomyia*) *splendida* Bonne-Wepster & Bonne 1919 | MB10217, MB10585, MB10652, MB10653, MB10655 | M/F/L |
| *Wyeomyia* (*Spilonympha*) *bourrouli* (Lutz 1905) | MB10701, ST10250 | F/L |
| *Wyeomyia* (*Triamya*) *aporonoma* Dyar & Knab 1906 | MB10689, MB10690, MB10691 | L |
| *Wyeomyia* (*Wyeomyia*) *pertinans* (Williston 1896) | MB10004, MB10007, MB10031, MB10033, MB10151, MB10189, MB10316, MB10317, MB10318, MB10385, MB10503 | M/F/L |
| *Wyeomyia* (*Wyeomyia*) *arthrostigma* (Lutz 1905) | MB10347, MB10348, MB10467, MB10528, MB10631 | M/F/L |
| *Wyeomyia* (*Wyeomyia*) *robusta* Sevenet & Abonnenc 1939 | MB10034, MB10035, MB10307, MB10386, MB10537 | M/F/L |
| *Wyeomyia* (Uncertain) *albosquamata* Bonne-Wepster & Bonne 1919 | MB10100, MB10101, MB10102, MB10155, MB10586 | M/F/L |
| *Wyeomyia* (Uncertain) *argenteorostris* (Bonne-Wepster & Bonne 1919) | MB10509, MB10619, MB10620, MB10621 | M/F/L |
| *Wyeomyia* (Uncertain) *compta* Senevet & Abonnenc 1939 | MB10490, MB10531, MB10535 | M/F/L |
| *Wyeomyia* (Uncertain) *melanocephala* Dyar & Knab 1906 | MB10264, MB10262, MB10263, MB10322, MB10393 | M/F/L |
| *Wyeomyia* (Uncertain) *occulta* Bonne-Weptser & Bonne 1919 | MB10270, MB10271, MB10272, MB10432, MB10457, MB10522 | M/F/L |
| *Wyeomyia* (Uncertain) *surinamensis* Bruijning 1959 | MB10482, MB10483, MB10484, MB10555, MB10587 | M/F/L |
| *Wyeomyia* sp.stC | MB10589, MB10590, MB10630 | L |
| **Culicinae: Toxorhynchitini** |  |  |
| *Toxorhynchites* (*Lynchiella*) *guadeloupensis* (Dyar & Knab 1906) | MB10540, MB10638, MB10639, ST10266 | M/F/L |
| *Toxorhynchites* (*Lynchiella*) *haemorrhoidalis haemorrhoidalis* (Fabricius 1787) | MB10124, MB10126, MB10670, MB10671, MB10672 | M/F/L |
| *Toxorhynchites* (*Lynchiella*) *haemorrhoidalis superbus* (Dyar & Knab 1906) | MB10570, MB10673, MB10674, MB10675, MB10676, MB10677, MB10678, MB10679, MB10680, ST10004 | M/F/L |
| *Toxorhynchites* (*Lynchiella*) *moctezuma* (Dyar & Knab 1906) | ST10035 | M |
