# Supplementary material for: DNA reference libraries of French Guianese mosquitoes for barcoding and metabarcoding
Source: PLoS One. 2017 Jun 2;12(6):e0176993. doi: 10.1371/journal.pone.0176993 (PMC5456030; doi:10.1371/journal.pone.0176993)
Supplement: S2 Table — Taxa are listed alphabetically and ranked by subfamily, tribe, genus and subgenus. Distances (p-distance) correspond to the percentage of dissimilar pairwise nucleotides and counts correspond to the number of voucher specimens included in this study followed, between brackets, by the total number of specimens (including ours) present in the BOLD database. (DOCX) [file pone.0176993.s002.docx]

**S2 Table. List of Barcode Index Numbers (BINs) with their associated species or morphospecies (hereafter ‘taxa’) obtained from the Barcode of Life Data Systems (BOLD; last visited august 2016). Taxa are listed alphabetically and ranked by subfamily, tribe, genus and subgenus. Distances (p-distance) correspond to the percentage of dissimilar pairwise nucleotides and counts correspond to the number of voucher specimens included in this study followed, between brackets, by the total number of specimens (including ours) present in the BOLD database.**

| **BIN** | **Species/morphospecies** | **Average distance** | **Maximum distance** | **Nearest-neighbour distance** | **Count** |
| --- | --- | --- | --- | --- | --- |
| **Anophelinae** | |  |  |  |  |
| ACZ3766 | *An.* (*Ano.*) *eiseni* | 0.94 | 0.94 | 7.06 | 2 |
| ACZ4390 | *An.* (*Ker.*) *neivai* | 0 | 0 | 6.74 | 4 |
| **Culicinae: Aedini** | |  |  |  |  |
| ABW1628 | *Ae.* (*Gec.*) *fluviatilis* | 1.11 | 1.53 | 9.62 | 2(6) |
| ACZ4358 | *Ae.* (*How.*) *arborealis* | NA | NA | 9.00 | 1 |
| AAH9007 | *Ae.* (*Och.*) *scapularis* | 1.15 | 5.00 | 6.68 | 3(301) |
| AAN3110 | *Ae.* (*Och.*) *serratus* | 1.33 | 2.41 | 1.70 | 2(10) |
| AAA4210 | *Ae.* (*Stg.*) *aegypti* | 1.07 | 6.31 | 1.58 | 3(538) |
| AAU1467 | *Hg.* (*Hag.*) *janthinomys* | 1.09 | 1.99 | 7.13 | 3(12) |
| AAO0580 | *Ps.* (*Jan.*) *ferox* | 1.18 | 2.00 | 1.77 | 3(13) |
| **Culicinae: Culicini** | |  |  |  |  |
| AAG3837 | *Cx.* (*Car.*) *infoliatus/urichii* | 0.41 | 0.77 | 7.25 | 5(14) |
| ACZ3921 | *Cx.* (*Car.*) sp.stI | 0.16 | 0.16 | 4.51 | 2 |
| AAN3636 | *Cx.* (*Cux.*) *coronator* | 0.66 | 2.95 | 1.17 | 5(136) |
| AAF1735 | *Cx.* (*Cux.*) *mollis* | 1.45 | 5.07 | 1.59 | 3(294) |
| AAA4751 | *Cx.* (*Cux.*) *quinquefasciatus* | 0.14 | 5.01 | 0.98 | 2(3314) |
| ABX7935 | *Cx.* (*Mcx.*) *imitator* | 0.61 | 0.92 | 4.17 | 2(4) |
| ACZ4187 | *Cx.* (*Mcx.*) *pleuristriatus* | 0.29 | 0.61 | 7.71 | 2 |
| ACZ3799 | *Cx.* (*Mcx.*) *stonei* | 0.94 | 0.94 | 8.23 | 2 |
| ACZ4071 | *Cx.* (*Mcx.*) *stonei* | 0.20 | 0.31 | 4.65 | 3 |
| ACZ4175 | *Cx.* (*Mcx.*) *stonei* | NA | NA | 4.01 | 1 |
| ACZ4194 | *Cx.* (Uncertain) *nigrimacula* | 0 | 0 | 8.45 | 3 |
| ACZ4158 | *Cx.* (Uncertain) *ocellatus* | 0.42 | 0.92 | 8.30 | 5 |
| ACZ4398 | *Culex* sp.stJ | 0.41 | 0.61 | 11.25 | 3 |
| ACZ4266 | *Culex* sp.stK | 0 | 0 | 2.89 | 2 |
| ACZ3899 | *Culex* sp.stL | NA | NA | 9.63 | 1 |
| AAW1435 | *Lt.* (*Lut.*) *allostigma* | 0.12 | 0.31 | 5.62 | 2(5) |
| **Culicinae: Orthopodomyiini** | |  |  |  |  |
| ACZ4163 | *Or. fascipes* | 0.16 | 0.16 | 2.41 | 2 |
| **Culicinae: Sabethini** | |  |  |  |  |
| ACZ4070 | *Jb. longipes* | 0.62 | 1.83 | 8.24 | 6 |
| ACZ4300 | *Jb. ulopus* | 0.62 | 0.62 | 9.21 | 2 |
| ACN9473 | *Li. durhamii* | 0.13 | 0.31 | 1.12 | 5(6) |
| AAW1293 | *Li. flavisetosus* | 0.03 | 0.15 | 7.54 | 3(10) |
| ACN0508 | *On.* sp.stA | 0.13 | 0.31 | 11.08 | 5(6) |
| ACZ3754 | *Ru.* (*Cte.*) *magna* | 0.10 | 0.15 | 2.57 | 3 |
| ACZ3755 | *Ru.* (*Cte.*) *magna* | 0.76 | 0.76 | 2.57 | 2 |
| AAW5410 | *Sa.* (*Pey.*) *undosus* | 0.10 | 0.31 | 3.53 | 5(6) |
| ACZ3825 | *Sa.* (*Pey.*) *hadrognathus* | NA | NA | 5.94 | 1 |
| ACZ3826 | *Sa.* (*Pey.*) *hadrognathus* | 0.46 | 0.46 | 5.94 | 2 |
| ACZ3779 | *Sa.* (*Pey.*) *paradoxus* | 0 | 0 | 8.19 | 2 |
| ACZ3827 | *Sa.* (*Pey.*) *soperi* | 0 | 0 | 8.99 | 3 |
| ACZ3811 | *Sa.* (*Pey.*) sp.stD | NA | NA | 9.47 | 1 |
| ACZ3883 | *Sa.* (*Sab.*) *cyaneus* | NA | NA | 7.88 | 1 |
| ACZ4359 | *Sa.* (*Sab.*) sp.stE | 0 | 0 | 1.77 | 2 |
| ACZ3810 | *Sa.* (*Sab.*) sp.stM | NA | NA | 8.84 | 1 |
| ACZ3828 | *Sa.* (*Sbn.*) *idiogenes* | 0 | 0 | 3.21 | 3 |
| ACZ4350 | *Sa.* (*Sbn.*) sp.stF | 0 | 0 | 3.21 | 2 |
| ACZ4319 | *Sh. fluviatilis* | NA | NA | 3.70 | 1 |
| ACZ4320 | *Sh. fluviatilis* | NA | NA | 3.70 | 1 |
| ACZ3895 | *Sh. schedocyclia* | 0 | 0 | 1.93 | 2 |
| ACZ3896 | *Sh. schedocyclia* | 0.10 | 0.15 | 1.93 | 3 |
| ACZ3752 | *Tr. compressum* | NA | NA | 6.90 | 1 |
| AAG3842 | *Tr. digitatum* | 0.23 | 0.46 | 2.57 | 1(4) |
| ACZ3792 | *Tr. digitatum* | 0 | 0 | 2.57 | 2 |
| ACZ3837 | *Tr. pallidiventer* | NA | NA | 7.06 | 1 |
| ACZ3838 | *Tr. pallidiventer* | 0 | 0 | 7.06 | 2 |
| ACZ4400 | *Trichoprosopon* sp.stG | NA | NA | 8.51 | 1 |
| ACZ4399 | *Trichoprosopon* sp.stH | NA | NA | 6.90 | 1 |
| ACZ4113 | *Wy.* (*Cae.*) sp.stB | 1.07 | 1.07 | 8.52 | 2 |
| ACZ3978 | *Wy.* (*Cru.*) *forattinii* | 0.12 | 0.31 | 8.35 | 5 |
| AAG3839 | *Wy.* (*Dec.*) *pseudopecten* | 1.38 | 2.45 | 4.49 | 4(7) |
| ACZ4104 | *Wy.* (*Dec.*) *pseudopecten* | 0.29 | 0.78 | 5.24 | 6 |
| ACA0978 | *Wy.* (*Den.*) *complosa* | 1.63 | 3.06 | 7.88 | 5(14) |
| ACZ3898 | *Wy.* (*Den.*) *luteoventralis* | 1.36 | 3.06 | 4.49 | 9 |
| ACZ3881 | *Wy.* (*Den.*) *testei* | 0.13 | 0.31 | 8.17 | 7 |
| ACZ4034 | *Wy.* (*Den.*) *ypsipola* | 0.22 | 0.32 | 7.50 | 3 |
| ACZ4140 | *Wy.* (*Dod.*) *aphobema* | 0.46 | 0.46 | 2.73 | 2 |
| ACZ3890 | *Wy.* (*Hys.*) *lamellata* | 0.16 | 0.31 | 4.29 | 4 |
| ACZ3891 | *Wy.* (*Myamyia*) *oblita* | 0 | 0 | 5.94 | 2 |
| ACZ4220 | *Wy.* (*Pho.*) *splendida* | 0.34 | 0.61 | 7.38 | 5 |
| ACZ4080 | *Wy.* (*Spi.*) *bourrouli* | 0.15 | 0.15 | 8.67 | 2 |
| ACZ4142 | *Wy.* (*Triamyia*) *aporonoma* | 0 | 0 | 3.05 | 3 |
| ACZ3855 | *Wy.* (*Wyo.*) *arthrostigma* | NA | NA | 2.29 | 1 |
| ACZ3856 | *Wy.* (*Wyo.*) *arthrostigma* | 0.00 | 0.00 | 2.29 | 4 |
| ACZ4079 | *Wy.* (*Wyo.*) *pertinans* | 0.45 | 1.68 | 1.44 | 11(12) |
| ACZ3847 | *Wy.* (*Wyo.*) *robusta* | 0.31 | 0.62 | 10.48 | 5 |
| ACZ4171 | *Wy.* (Uncertain) *albosquamata* | 0.06 | 0.16 | 9.19 | 5 |
| ABW3718 | *Wy.* (Uncertain) *argenteorostris* | 0.16 | 0.31 | 11.56 | 4(6) |
| ACZ4141 | *Wy.* (Uncertain) *compta* | 0 | 0 | 3.08 | 3 |
| ACZ3830 | *Wy.* (Uncertain) *melanocephala* | 0.21 | 0.46 | 2.25 | 5 |
| ACZ4130 | *Wy.* (Uncertain) *occulta* | 0.36 | 0.48 | 9.66 | 6 |
| ACZ4312 | *Wy.* (Uncertain) *surinamensis* | 0.12 | 0.31 | 7.20 | 5 |
| ACZ4143 | *Wyeomyia* sp.stC | 0.10 | 0.15 | 10.00 | 3 |
| **Culicinae: Toxorhynchitini** | |  |  |  |  |
| ACZ4355 | *Tx.* (*Lyn.*) *guadeloupensis* | 0.31 | 0.61 | 8.26 | 4 |
| ACZ4120 | *Tx.* (*Lyn.*) *haemorrhoidalis haemorrhoidalis* | 0.49 | 0.77 | 7.04 | 5 |
| ACZ3913 | *Tx.* (*Lyn.*) *haemorrhoidalis superbus* | 0.25 | 0.47 | 1.44 | 6 |
| ACZ3996 | *Tx.* (*Lyn.*) *haemorrhoidalis superbus* | 0 | 0 | 1.44 | 3 |
| ACZ4119 | *Tx.* (*Lyn.*) *haemorrhoidalis superbus* | NA | NA | 6.38 | 1 |
| ACZ4278 | *Tx.* (*Lyn.*) *moctezuma* | NA | NA | 8.08 | 1 |
